# Curated Collections for Educators: Five Key Papers on Evaluating Digital Scholarship

**DOI:** 10.7759/cureus.2021

**Published:** 2018-01-03

**Authors:** Antonia Quinn, Teresa M Chan, Christopher Sampson, Catherine Grossman, Christine Butts, John Casey, Holly Caretta-Weyer, Michael Gottlieb

**Affiliations:** 1 Department of Emergency Medicine, SUNY Downstate College of Medicine; 2 Faculty of Health Sciences, Department of Medicine, Division of Emergency Medicine, McMaster University; 3 Emergency Medicine, University of Missouri Columbia; 4 Pulmonary and Critical Care Medicine, Virginia Commonwealth University Health Systems; 5 Section of Emergency Medicine, Louisiana State University Health Sciences; 6 Department of Emergency Medicine, Ohiohealth Doctors Hospital; 7 Department of Emergency Medicine, Oregon Health & Science University; 8 Department of Emergency Medicine, Rush University Medical Center

**Keywords:** curated collection, digital scholarship evaluation, academic promotion, medical education

## Abstract

Traditionally, scholarship that was recognized for promotion and tenure consisted of clinical research, bench research, and grant funding. Recent trends have allowed for differing approaches to scholarship, including digital publication. As increasing numbers of trainees and faculty turn to online educational resources, it is imperative to critically evaluate these resources. This article summarizes five key papers that address the appraisal of digital scholarship and describes their relevance to junior clinician educators and faculty developers.

In May 2017, the Academic Life in Emergency Medicine Faculty Incubator program focused on the topic of digital scholarship, providing and discussing papers relevant to the topic. We augmented this list of papers with further suggestions by guest experts and by an open call via Twitter for other important papers. Through this process, we created a list of 38 papers in total on the topic of evaluating digital scholarship. In order to determine which of these papers best describe how to evaluate digital scholarship, the authorship group assessed the papers using a modified Delphi approach to build consensus.

In this paper we present the five most highly rated papers from our process about evaluating digital scholarship. We summarize each paper and discuss its specific relevance to junior faculty members and to faculty developers. These papers provide a framework for assessing the quality of digital scholarship, so that junior faculty can recommend high-quality educational resources to their trainees. These papers help guide educators on how to produce high quality digital scholarship and maximize recognition and credit in respect to receiving promotion and tenure.

## Introduction and background

The manifestation of scholarship is changing. Whereas traditionally, academia only recognized clinical research, benchwork, peer reviewed publications, and grant funding as markers of success, other forms of scholarship were defined in the late 20th century by Ernest L. Boyer. Dr. Boyer, through his seminal work, Scholarship Reconsidered, created a major paradigm shift to include other forms of scholarship, namely scholarship of integration, scholarship of application/engagement, and scholarship of teaching and learning [1].

With the advent of the digital age, however, disruptive technologies like social media are now pushing us even closer towards yet another paradigm shift. In the age of the printing press, publishing and distributing ground-breaking new ideas were controlled by publishing houses. In today’s age, the Web 2.0 applications now allow for ease of publication at an unprecedented level. This has led to a veritable explosion of certain types of digital products, including blogs, podcasts, and tweet chats [2-4].

However, recent trends have allowed for differing approaches to scholarship, including digital publication [5-6]. While there is a compelling case that these new forms of scholarship are actually no different from prior technologies (e.g. Is a blog post not just the modern interpretation of a text book? Isn’t a podcast just a more easily distributed recording of a lecture?), their ease of digital publication makes quality surveillance imperative [7].

As increasing numbers of trainees and faculty turn to online educational resources, it is imperative to evaluate digital resources for quality. Trainees and practicing physicians alike are inconsistent in gestalt recommendations of online educational content [8-9]. In order for academia to accept these disruptive forms of scholarship, it is imperative these publications be scrutinized and rigorously assessed for content and quality in the same way as reserved for traditional scholarship.

In 2017, the Faculty Incubator was created by the Academic Life in Emergency Medicine (ALiEM) team to create a community of practice (CoP) for early career educators. In this online forum, members of this CoP discuss and debate topics relevant to modern clinician educators. To that end, we created a one month module focused on learning technologies, with digital scholarship as a prominent point of discussion.

Since there is an emerging literature base on this important topic, our team sought to identify key literature about how to evaluate the quality of digital scholarship. This paper utilized a consensus-based review process to determine which papers may assist junior educators who wish to learn more about how they can evaluate their digital scholarship. These papers provide a framework for assessing digital scholarship for quality so that junior faculty can recommend quality educational resources to their trainees. Furthermore, by highlighting the parts of digital scholarship that produce high quality, future writers of online content may be able to better shape their work in order to maximize credit when applying for promotion and tenure.

## Review

Methods

In the first month of the ALiEM 2017-2018 Faculty Incubator, we discussed the topic of digital scholarship. We monitored the proceedings of this CoP from May 1st-31st, 2017. The monitored online discussions involved both junior faculty members and senior faculty mentors. While discussions occurred, we gathered the titles of papers that were cited, shared, and recommended within our online discussion forum and compiled these into a list.

This list was then expanded via two other methods. First, we consulted two content experts, Brent Thoma and Jonathan Sherbino, for suggestions. Next, we posted a call for important papers regarding the evaluation of digital scholarship on Twitter. We ‘tweeted’ requests to have participants of the #FOAMed and #MedEd online communities provide suggestions for important papers on the topic of peer review.

Once the augmented list was completed, we conducted a three-round voting process inspired by the Delphi methodology. Similar methods were used on our previous papers to build consensus on the most important papers to feature [10-13] [14-16]. Readers will note that this was not traditional Delphi methodology because our raters included novices (i.e. junior faculty members, participants in the Faculty Incubator), as well as experienced/expert medical educators (i.e. clinician educators, all of whom have published >10 peer reviewed publications, who serve as mentors and facilitators of the ALiEM Faculty Incubator). Rather than only including experts, we intentionally involved junior educators to ensure we selected papers that would be of use to a spectrum of educators throughout their careers.

Results

Our Faculty Incubator discussions yielded 37 papers, and the expert recommendations and social media calls yielded a total of one additional article. Our process provides a rank-order listing of all these papers in the order of perceived relevance, from the most to the least relevant. Our top five papers are expanded upon below. After our third round of voting, we had a tie for the final article leading to a fourth round of voting. We included the article not chosen as the fifth paper as an 'honorable mention'. Our ratings of all 38 papers are listed in Table 1, along with their full citations.

**Table 1:** The complete list of 38 literature items collected by the authorship team

Citation	ROUND 1. Initial mean scores (SD). Max score 7	ROUND 2. % of raters that endorsed this paper	ROUND 3. % of raters that endorsed this paper	ROUND 4. Tie break round	Top 5 papers
Cabrera, et al. [6]	6.6(0.52)	100%	87.5%		1
Thoma, et al. [17]	5.4(1.30)	87.5%	87.5%		1
Chan TM, et al. [18]	6.3(0.89)	87.5%	75.0%		2
Colmers, et al. [19]	5.8(1.39)	87.5%	75.0%		2
Sherbino, et al. [5]	6.0(1.41)	75%	50.0%	62.5%	5
Gottlieb, et al. [20]	5.8 (0.71)	87.5%	50.0%	37.5%	Honorable Mention
Thoma B, et al. [21]	5.8 (1.28)	50%	0%		
Chan T, et al. [22]	5.5(1.20)	50%	0%		
Frank JR, et al. [23]	5.5(1.69)	50%	12.5%		
Cameron CB, et al. [24]	5.3(1.49)	75%	12.5%		
Lin M, et al. [25]	5.3 (1.49)	75%	12.5%		
Krishnan K, et al. [8]	5.1(1.49)	62.5%	37.5%		
Chan TM, et al. [26]	5.0(1.69)	50%	0%		
Thoma B, et al. [27]	5.0(2.07)	25%	0%		
Lin M, et al. [28]	4.9(1.13)	62.5%	0%		
Flynn L, et al. [29]	4.9(1.25)	37.5%	0%		
Sherbino J, et al. [30]	4.9(1.64)	37.5%	0%		
Boyer EL. [1]	4.6 (2.33)	50%	0%		
Sterling M, et al. [31]	4.6 (1.19)	25%	0%		
Purdy E, et al. [32]	4.5 (0.76)	12.5%	0%		
Paterson QS, et al. [33]	4.4 (1.69)	12.5%	0%		
Eysenbach G [34]	4.4 (1.51)	50%	0%		
Roland D, et al. [35]	4.1 (1.55)	0	0%		
Lumba-Brown A, et al. [36]	4.1 (0.83)	12.5%	0%		
Jordan J, et al. [37]	4.0 (1.93)	0	0%		
Boulos MN, et al. [38]	4.0 (1.31)	12.5%	0%		
Sutherland S, et al. [39]	3.9 (1.64)	12.5%	0%		
Diug B, et al. [40]	3.9 (0.83)	0	0%		
Lin M, et al. [41]	3.8 (0.89)	12.5	0%		
Raine T, et al. [42]	3.8 (1.83)	0	0%		
Riddell J, et al. [43]	3.8 (0.89)	0	0%		
Thoma B, et al. [44]	3.6 (1.60)	0	0%		
Ke Q, et al. [45]	3.4 (2.13)	0	0%		
Hillman T, et al. [46]	3.4 (1.60)	0	0%		
Pronk NP, et al. [47]	3.0 (2.39)	0	0%		
Flynn S, et al. [44]	3.0 (1.31)	0	0%		
Solomon DJ, et al. [48]	2.8 (1.58)	0	0%		
Langdorf MI, et al. [49]	2.6 (1.77)	0	0%		

Discussion

The following is a list of papers that our group has determined to be of interest and relevance to junior faculty members and more senior colleagues who may be in charge of faculty development. The accompanying commentaries are meant to explain the relevance of these papers to junior faculty members and also highlight considerations for senior faculty members when using these works for faculty development workshops or sessions.

*1. Thoma B, Chan T, Benitez J, Lin M. Educational Scholarship in the Digital Age: A Scoping Review and Analysis of Scholarly Products, The Winnower 1: e141827. 77297, 2014, DOI: 10.15200/winn *[17].

Summary: This article applies the more widely accepted Boyer model of scholarship to classify digital works [1]. A literature review and coding schema allow the different forms of digital media presented to be reclassified into Boyer’s four subtypes of scholarship: teaching, integration, application, and discovery [1]. Various types of digital scholarship were also reviewed and summarized. Over 85% of digital scholarship was mapped to the teaching subtype, although some mapped to more than one subtype. The weakness most associated with digital scholarship was ensuring rigorous scrutiny for quality, which is presumed to occur with traditional scholarship. A framework, such as the one proposed, is valuable as the development and distribution of digital scholarship continues to increase and these works are integrated into academic portfolios.

Relevance to junior faculty members: Associating various types of digital scholarship with a well-established and accepted education framework allows junior faculty to strengthen their academic portfolio. Demonstrating how their digital works fit into the established subtypes of Boyer’s scholarship framework can assist junior faculty when explaining their work to a promotion and tenure committee. This may be particularly valuable by normalizing digital scholarship into terms that promotion and tenure committees may be more familiar with.

Relevance to faculty developers: Thoma and colleagues go beyond demonstrating how digital scholarship can fit into the framework of Boyer’s four subtypes of scholarship to make a case for developing tools that can examine the impact and quality of these digital products [17]. The growing number of digital products identified in the literature emphasizes the increased use of digital technology for educational scholarship with most being categorized into Boyer’s scholarship of teaching and learning [1]. The authors concluded that there is no compelling evidence that digital products are more effective but, given their widespread use, further research is needed in order to assess their quality.

*2. Cabrera D, Vartabedian BS, Spinner RJ, Jordan BL, Aase LA, Timimi FK. More Than Likes and Tweets: Creating Social Media Portfolios for Academic Promotion and Tenure. Journal of Graduate Medical Education. 2017 Aug;9(4):421-5 *[6].

Summary: More education faculty are focusing their scholarly efforts on the creation, curation, and dissemination of free, open-access medical education. In this paper, Cabrera and colleagues propose a framework to incorporate social media scholarship into current academic promotion and tenure systems. They provide recommendations of best practices for institutions in implementing a system for recognizing social media and digital scholarship for academic promotion. This includes the development of focused guidelines and strategic priorities for social media use, the development of a clear impact grid to identify types of social media activities considered for academic tenure, and methods by which the quality and impact of scholarship can be measured. Suggested measures of impact include altmetrics such as page views, peer review of the work, and objective measures of quality such as those laid out by Sherbino and colleagues [5]. An example impact grid is also included in the paper. Faculty scholars are encouraged to create and maintain a social media scholarship portfolio, much the same as they would an educator’s portfolio for their work in clinical and didactic teaching, curriculum design, and formal mentorship. The description of one’s scholarly work in social media should remain consistent with Glassick’s framework and include clear goals, adequate preparation, appropriate methods, significant results, effective presentation, and reflective technique [50].

Relevance to junior faculty members: This paper lays out a clear framework for presenting social media and digital scholarship for academic promotion and tenure. Junior faculty should develop and curate a social media scholarship portfolio, which includes a statement of social media scholarship philosophy, academic niche, audience, objectives, and platforms. All aspects of social medial scholarship should be presented and include original content creation, curation of content, community management, platform administration, data analysis, and a durable record of scholarship (eg, permalinks, cached content). A clear description of how social media scholarship aligns with the junior faculty member’s overall career development plan and program of scholarship will provide a cohesive picture for the promotion and tenure committee at the time of review.

Relevance to faculty developers: This is an essential paper for educators who engage in social media to receive proper recognition when applying for promotion and tenure. The authors outline a list of potential social media scholarship avenues, as well as their relative impact. It also provides a guideline for developing local institutional criteria for social media-based scholarship in addition to a formal definition based upon the paper by Sherbino and colleagues [5]. This resource may be provided to one’s academic appointment committee when applying for promotion, but would be better utilized in advance to help develop local criteria.

*3. Colmers IN, Paterson QS, Lin M, Thoma B, Chan TM. The Quality Checklists for Health Professions Blogs and Podcasts, The Winnower 2: e144720. 08769, 2015, DOI: 10.15200/winn. 144720.08769 Colmers, et al. This article is distributed under the terms of the Creative Commons Attribution.;4 *[19].

Summary: Blogs and podcasts are highly popular in medical education and have grown exponentially without significant oversight or quality control. This paper seeks to create an easy-to-use checklist for educators to evaluate and identify the educational value of a blog or podcast. To do so, the authors used a three-step process. Initially, they performed a literature review with the goal of extracting quality indicators of secondary educational resources. These findings then underwent qualitative analysis to isolate those that would be applicable to blogs or podcasts, yielding a total of 151 indicators. To further refine these quality indicators, the authors then surveyed a large and varied group of expert producers of online medical education content to determine which of this initial group of indicators was most important. This analysis yielded a more manageable 14 indicators for blogs and 26 for podcasts. To avoid bias in surveying only experts in content development, the authors also surveyed general medical education experts. From the initial group of 151 quality indicators, the general medical experts identified three indicators for blogs, one for podcasts, and nine for both blogs and podcasts. Once these surveys were completed, the authors compiled the findings from both the expert producers of online content and the general medical education experts to form simplified checklists for both blogs and podcasts. The checklists are similarly divided into three sections (ie, credibility, content, and design) and contain a series of questions that can be answered yes, no, or unclear.

Relevance to junior faculty members: Many educators and students enjoy blogs and podcasts due to their entertainment value, brevity, and style. They offer a welcome break from more traditional methods such as textbooks or lectures. However, there is a tendency to overlook the content and quality of a blog or podcast when blinded by other factors, such as the aforementioned entertainment value. This checklist offers junior faculty, who may have limited experience in evaluating online educational materials, a simple and rapid method to assess the worth of a blog or podcast as a teaching tool. By focusing on key aspects of the blog or podcast, this checklist ensures that the junior faculty will be able to accurately assess its quality.

Relevance to faculty developers: This paper is invaluable for faculty development for both consumers and producers of digital scholarship. Assessing the quality of podcasts and blogs based on personal gestalt has been shown to be unreliable [8]. This paper provides an easily usable checklist for developers, editors, and end-users of blogs and podcasts. This checklist helps guide the producer to focus their efforts to produce a quality product. Editors can utilize rubric to provide quality feedback to writers to optimize the peer review process, advancing the overall quality of digital scholarship. Educators can guide learners to critically appraise blogs and podcasts prior to their use.

*4. Chan TM, Grock A, Paddock M, Kulasegaram K, Yarris LM, Lin M. Examining Reliability and Validity of an Online Score (ALiEM AIR) for Rating Free Open Access Medical Education Resources. Annals of Emergency Medicine. 2016 Dec 31;68(6):729-35 *[18].

Summary: This article compared a global rating gestalt-based Likert scale versus a five-domain rating scale (ALiEM approved instructional resources score (AIR Score)) to evaluate free open access medical education (FOAM) blog posts. The AIR Score included ratings of: Best Evidence in Emergency Medicine rater scale, content accuracy, educational utility, having an evidence-based medicine construct, and provision of author and literature references. The AIR score had moderate congruence with the gestalt evaluations and required nine raters to achieve adequate interrater reliability per article.

Relevance to junior faculty members: This article is important to junior faculty as it provides an initial scaffold for evaluating existing resources, as well as a framework for creating quality online FOAM resources. As evaluation tools for FOAM resources evolve, it will be important to keep up with literature to adapt publications as needed to stay within best practices.

Relevance to faculty developers: This paper is of two-fold importance for those interested in faculty development. First, it is a good example of applying measurement science to an educationally-relevant scoring system. This can open up a discussion around the methodology used by the authors to generate ‘validity evidence’ for a particular scoring system, which can be a nice way to compare and contrast with known diagnostic test validation methods. Moreover, it is an example of how you can generate multiple wins from a single educational innovation [20]. Notably, this group has authored a paper describing their innovation [28] and subsequently analyzed the data they acquired whilst running their innovation processes to generate validity evidence around the scoring system they created.

*5. Sherbino J, Arora VM, Van Melle E, Rogers R, Frank JR, Holmboe ES. Criteria for Social Media-based Scholarship in Health Professions Education. Postgraduate Medical Journal. 2015 Oct 1;91(1080):551-5 *[5].

Summary: Sherbino and colleagues recognized that substantial questions were present on how social media-based scholarship was viewed through traditional scholarship assessment methods [5]. Using a consensus approach, they were able to define the criteria for social media-based scholarship. Following a facilitated session of both in-person and virtual health professions educators, a consensus statement was produced. This statement required that social media-based scholarship in health professions must: be original, advance the field of health professions education, be archived and disseminated, and provide the community the ability to comment on and provide feedback in a transparent fashion. A process was also defined that listed the standards of criteria for authorship. With respect to the impact on education, the authors agree that evidence of a transparent critical appraisal is required. Also, the innovations must have the potential to impact health professions in a rapid or broad fashion. Ease of accessibility is also a concern. Alternative metrics were suggested as a way to demonstrate the dissemination and impact of social media-based scholarship. They concluded that the health professions education community should champion social media-based scholarship as a legitimate educational pursuit.

Relevance to junior faculty members: Given the use of social media-based educational scholarship among junior faculty, it is important to know criteria that can be applied in order to make one’s scholarship more robust and of high quality. This enables a junior faculty member’s productivity to fit criteria that can help them when viewed by evaluators for academic advancement.

Relevance to faculty developers: With increasing use of social media within academic medicine, it is essential that junior faculty understand what constitutes scholarship. This paper builds upon the work of Glassick to define scholarship with social media and is complementary to the above paper by Cabrera [6, 50]. This may be a valuable resource to ensure that one’s efforts meet the criteria for scholarship in order to properly categorize and support projects when applying for promotion and tenure. It is equally important to ensure that faculty understand what does not constitute scholarship, so as to realign one’s efforts with a project that meets the criteria and may maximize their efforts [20].

*Honorable Mention: Gottlieb M, Chan TM, Sherbino J, Yarris L. Multiple Wins: Embracing Technology to Increase Efficiency and Maximize Efforts. AEM Education and Training *[20].

This paper was highly-ranked by our team, but ultimately not selected because it does not bear weight on how to actually evaluate digital scholarship. Instead, we summarize it here because we feel that it is a valuable “how-to” article that may guide how junior faculty members align projects and harness the power of technology to efficiently increase their scholarly efforts [20]. While it does not help those seeking evaluative tools for judging the quality of digital scholarship, it describes well how one might integrate digital scholarship into their practice. 

Limitations

Many of the final articles share one or more of the authorship group of this manuscript. These same authors served as senior mentors and content experts for the month covering digital scholarship for the Aliem Faculty Incubator and therefore were essential in the writing of this manuscript. As with more systematic reviews, individuals with a good grasp of key literature and content experts, such as active researchers in a particular field, are best served to identify papers.

We attempted to minimize selection bias by “tweeting” requests for additional articles covering the evaluation of digital scholarship as well as multiple rounds of the modified Delphi. The chosen articles not only had to cover the topic of evaluating digital scholarship, they had to be beneficial for junior faculty. Articles not chosen via the modified Delphi methodology may not have been appropriate for junior faculty.

## Conclusions

This paper describes five key articles on the evaluation of digital scholarship. We believe this resource will be valuable for clinician educators to evaluate digital scholarship, while also providing guidance for producers of digital content to create a robust product that meets the definition of scholarship.
